# *Ganoderma triterpenes* Protect Against Hyperhomocysteinemia Induced Endothelial-Mesenchymal Transition via TGF-β Signaling Inhibition

**DOI:** 10.3389/fphys.2019.00192

**Published:** 2019-03-05

**Authors:** Jinzhao He, Yi Sun, Yingli Jia, Xiaoqiang Geng, Ruoyun Chen, Hong Zhou, Baoxue Yang

**Affiliations:** ^1^Key Laboratory of Molecular Cardiovascular Sciences, State Key Laboratory of Natural and Biomimetic Drugs, Department of Pharmacology, School of Basic Medical Sciences, Peking University, Beijing, China; ^2^State Key Laboratory of Bioactive Substance and Function of Natural Medicines, Chinese Academy of Medical Sciences, Institute of Materia Medica, Peking Union Medical College, Beijing, China

**Keywords:** homocysteine, endothelial cells, endothelial-mesenchymal transition, TGF-β, oxidative stress, *Ganoderma triterpenes*

## Abstract

Endothelial dysfunction is one of the most important pathological status in hyperhomocysteinemia (HHcy) related cardiovascular diseases. Whereas, the underlying mechanisms have not been fully elucidated yet, concomitant with the absence of effective treatment. The purpose of this study was to explore the main mechanisms involved in HHcy-induced endothelial injury and identify the protective effect of *Ganoderma triterpenes* (GT). Bovine aortic endothelial cells (BAECs) were applied as *in vitro* experimental model. The small molecular inhibitors were used to explore the signalings involved in HHcy-induced endothelial injury. The experimental results provided initial evidence that HHcy led to endothelial-mesenchymal transition (EndMT). Meanwhile, TGF-β/Smad, PI3K/AKT and MAPK pathways were activated in this process, which was demonstrated by pretreatment with TGF-β RI kinase inhibitor VI SB431542, PI3K inhibitor LY294002, p38 inhibitor SB203580, and ERK inhibitor PD98059. Furthermore, it was found that GT restrained the process of HHcy-induced EndMT via reducing oxidative stress and suppressing fore mentioned pathways with further inhibiting the activity of Snail. These results implicate that there is an untapped potential for GT as a novel therapeutic candidate for HHcy-induced EndMT through alleviating oxidative stress and canonical TGF-β/Smad and non-Smad dependent signaling pathways.

## Introduction

Cardiovascular diseases (CVDs) have severely threatened human health in light of high morbidity and mortality. Hyperhomocysteinemia (HHcy) has been considered as an established independent risk factor for CVDs, such as hypertension, atherosclerosis, heart failure, and stroke (Pushpakumar et al., 2013; Spence et al., 2017). Previous studies have proven that increasing plasmatic Hcy is positively associated with the severity of CVD (Hankey and Eikelboom, 1999; Shenoy et al., 2014). The pathophysiology of HHcy-mediated CVDs is characterized by vascular remodeling, lipid metabolism disorder and endothelial dysfunction (Steed and Tyagi, 2011; Lai and Kan, 2015).

HHcy-induced endothelial dysfunction is mainly involved in oxidative stress, inflammation, endoplasmic reticulum (ER) stress, and apoptosis (Lai and Kan, 2015; Wu et al., 2015). Oxidative stress has been reported to promote endothelial-mesenchymal transition (EndMT) in atherosclerosis, diabetic nephropathy and systemic sclerosis, which indicates the close relationship between oxidative stress and EndMT (Evrard et al., 2016; Ma et al., 2017; Thuan et al., 2018). ECs undergoing EndMT are typified by the loss of endothelial properties and the acquisition of mesenchymal cell phenotype. Blockage of EndMT through inhibiting the process of oxidative stress has been considered as a promising way to protect from endothelial injury.

*Ganoderma triterpenes*, the main component and bioactive metabolite of *Ganoderma lucidum*, have anti-oxidative activity. With regard to CVD, GT have been indicated to show potential cardiovascular protection on related animal models through anti-oxidation to prevent atherosclerosis and heart injury. In one study, it has been demonstrated its protective effect on vascular endothelium in carotid-artery-ligation mouse model by inhibiting oxidative stress (Hsu et al., 2018). In the other study, it was also reported that GT play the role of cardioprotection mainly by scavenging cellular reactive oxygen species (ROS) (Kuok et al., 2013). In addition, Li et al. (2016) showed that a supercritical-CO_2_ extract of *G. lucidum* spores could suppress TGF-β-induced EMT in cholangiocarcinoma. Notwithstanding, it is still undetermined whether GT have the effect on HHcy-induced oxidative stress and EndMT, as well as the detailed mechanism.

In the present study, we evaluated the hypothesis that HHcy could induce EndMT and cause endothelial injury, on which GT might have a protective effect. We provide evidence that EndMT is involved in the process of HHcy-induced endothelial injury, which could be alleviated by GT treatment via reducing oxidative stress and curtailing canonical TGF-β/Smad and non-Smad dependent signalings to regulate Smad and Snail-mediated gene transcription. These results suggest a possible mechanism for HHcy-induced endothelial dysfunction and provide a novel therapeutic insight for GT.

## Materials and Methods

### Cell Culture

BAECs were given as a gift from Dr. Wang Xian’s laboratory, in Peking University Health Science Center, Beijing, China. BAECs were cultured in Dulbecco’s modified Eagle’s medium (Gibco) containing 10% fetal bovine serum (Invitrogen), 100 μg/ml streptomycin, and 100 U/ml penicillin at 5% CO_2_ and 37°C humidified incubator. After the confluence reaching to 70–80%, the cells were made quiescent in serum free medium for 12 h, then pretreatment with 40 μM ERK1/2 inhibitor PD98059 (Calbiochem), 10 μM p38 inhibitor SB203580 (Calbiochem), and 5 μM TGF-β RI kinase inhibitor VI SB431542 (Calbiochem), 5 μM PI3K inhibitor LY294002 (Selleck) or 6.25, 12.5, and 25 μg/ml GT which were all dissolved in DMSO for 1 h. BAECs were incubated with Hcy dissolved in DMEM containing 2% FBS for indicated time. BAECs from passages 4–9 were used in this study.

### Cell Viability Assay

The CCK-8 kit (Dojindo) was utilized to monitor the cytotoxicity of Hcy. BAECs were seeded in 96 wells plates (7 × 10^3^ cells/well). When the cells grew into approximately 80% confluence, they were cultivated with serum free medium for 24 h and were stimulated by various concentration of Hcy for another 24 h. 100 μl 5% CCK-8 solution diluted with DMEM containing 10% FBS were added into each well. After 2 h, the absorbance value was measured with microplate reader (Biotek, MQX200) at wavelength of 450 nm.

### Western Blot Analysis

In brief, undergoing lysis with RIPA lysis buffer (Thermo Fisher Scientific) containing 4% protease inhibitor cocktail (Roche Diagnostics), homogenization, and centrifugation, the extraction of cell lysate was used to quantitative analysis of total protein by BCA kit (Pierce Biotechnology). Aliquots of protein solution were resolved in SDS-PAGE and separated based on their molecular size via electrophoresis. The proteins were shifted to polyvinylidene difluoride membranes (PVDF, Amersham Biosciences) and blocked with 5% non-fat milk or 2% BSA for 2 h at room temperature. Then, the PVDF membranes were incubated with primary antibodies against VE-cadherin (Biodragon), α-SMA (Santa Cruz Biotechnology), vimentin (Cell Signaling Technology), TGF-β1 (Santa Cruz Biotechnology), PAI-1 (Biodragon), p-AKT1 (Abclonal), AKT1 (Santa Cruz Biotechnology), p-GSK-3β (Cell Signaling Technology), GSK-3β (Cell Signaling Technology), Snail (Cell Signaling Technology), p-ERK1/2 (Santa Cruz Biotechnology), p-Smad2/3 (Santa Cruz Biotechnology), Smad2 (Invitrogen), ERK2 (Santa Cruz Biotechnology), p-p38 (Cell Signaling Technology), p38 (Cell Signaling Technology), NF-κB p65 (Bioworld), p-NF-κB p65 (Cell Signaling Technology), COX-2 (Bioworld), GRP78 (Cell Signaling Technology), CHOP (Santa Cruz Biotechnology), Nox4 (Biodragon), and β-actin (Santa Cruz Biotechnology) overnight at 4°C. Secondary antibody, goat anti-rabbit IgG or goat anti-mouse IgG (Santa Cruz Biotechnology), was incubated for 45 min at room temperature. The proteins were developed by ECL kit (Applygen Technologies Inc) and exposed to X-ray films or visualized with a chemiluminescence detection system (Gene Company Limited, XRQ). These data were analyzed by Quantity-one software and showed the relative protein level compared to control group.

### Oxidative Stress Assessment

Cells were cultured in 60 mm plastic plates (5 × 10^4^ cells/ml), homogenized lysis solution was harvested and centrifuged at 12,000 rpm for 20 min at 4°C. The supernatant was used to evaluate the level of superoxide dismutase (SOD) through specific reagent kits (NJJC Bio) and Western blot. BAECs were planted in 12-well plastic plates. After Hcy and GT treatment, cells were washed with 0.01 M PBS for three times. Then, cells were incubated with 10 μM 2′-7′-Dichlorodihydroflurescein diacetate (DCFH-DA, Sigma) for 30 min at 37°C. DCFH-DA was washed out with PBS for three times and cell nucleus were labeled with hochest (Leagene) for 5 min at 37°C. The intensity of DCFH-DA fluorescence was measured (Thermo scientific, Varioskan Flash) and represented the level of intracellular ROS.

### Data Analysis

One-way ANOVA or Student’s *t* test with Prism 5.0 software was used for statistical analysis. All data were presented as mean ± SEM of three independent experiments and performed as histogram with scale bar. *P* < 0.05 was identified to be statistical significance for all tests.

## Results

### Hcy Induced EndMT With Little Cytotoxicity

**FIGURE 1:**
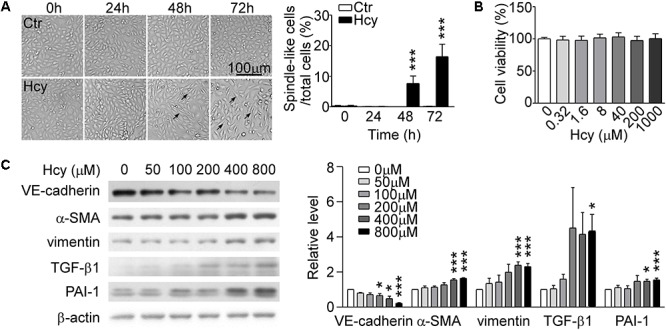
Hcy induced EndMT in BAECs. **(A)** BAECs were treated with or without Hcy (800 μM) for 0∼72 h, cell morphology was observed under phase contrast microscopy (*left*). Bar represents 100 μm. The arrows indicated the typical cells. Right panel shows the rate of spindle-like cells to total cells. **(B)** Cell viability of BAECs treated with Hcy (0∼1000 μM) for 24 h. **(C)** Representative Western blotting (*left*) showing the protein levels of VE-cadherin, α-SMA, vimentin, TGF-β1, and PAI-1. The quantification of protein levels (*right*) are shown in right panel. Data are mean ± SEM. *n* = 3; ^∗^*P* < 0.05; ^∗∗∗^*P* < 0.001 vs. control group.

By observing cell morphology, we found that BAECs lost their cobblestone appearance and presented elongated and spindle-like morphology after stimulating with 800 μM Hcy over 48 h compared with untreated cells (Figure 1A, left panel). The rate of spindle-like cells to total cells was counted for quantitative analysis (Figure 1A, right panel). Cell viability assay showed that Hcy had no cytotoxicity on BAECs within 0–1000 μM (Figure 1B). Western blot results exhibited that the expression of VE-cadherin reduced dose-dependently in Hcy-treated BAECs in comparison with control group (Figure 1C). The relative levels of mesenchymal cells and fibrosis marker α-SMA, vimentin, and TGF-β1 were elevated obviously in response to high dose of Hcy. The expression levels of PAI-1 in 400 and 800 μM Hcy-treated groups increased significantly compared with control group (Figure 1C). The results indicate that the cell-cell junction has been abated and the phenotype of BAECs has been altered in response to high concentration of Hcy.

### TGF-β/Smad and Non-Smad Dependent Signalings Were Involved in Hcy Mediated EndMT

**FIGURE 2:**
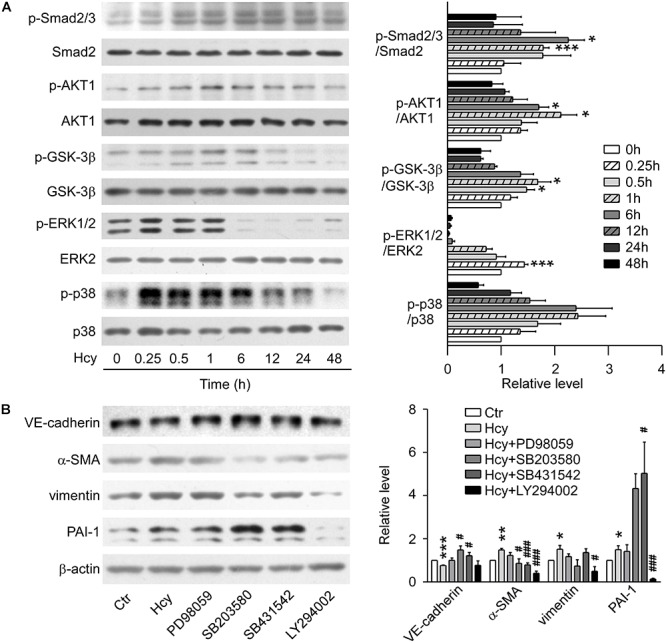
TGF-β/Smad, MAPK, and PI3K/AKT signalings were involved in Hcy-mediated EndMT. **(A)** Representative Western blotting (*left*) showing the time-course changes of p-Smad2/3, p-AKT, p-GSK-3β, p-ERK, and p-p38 protein levels in cells treated with 800 μM Hcy for 0–48 h. The quantification of protein levels are shown in right panel. **(B)** Representative Western blotting (*left*) showing the expression of VE-cadherin, α-SMA, vimentin, and PAI-1 in BAECs pre-incubated with PD98059, SB203580, SB431542 and LY294002 for 1 h, then treated with Hcy for 48 h (*left*). The quantification of protein levels are shown in right panel. Data are mean ± SEM. *n* = 3; ^∗^*P* < 0.05; ^∗∗^*P* < 0.01; ^∗∗∗^*P* < 0.001 vs. control group. ^#^*P* < 0.05; ^##^*P* < 0.01; ^###^*P* < 0.001 vs. Hcy-treated group.

In Western blot analysis, the expression of phosphorylated Smad2/3 was elevated and the protein level of ERK1/2 reached the peak at 15 min with Hcy treatment. Nevertheless, p38 MAPK signaling showed a tendency to be activated without significant statistical difference. The phosphorylation levels of AKT1 and its downstream molecule GSK-3β were significantly increased at 1 h with Hcy treatment, which suggests that the pathway of PI3K/Akt/GSK-3β was also activated (Figure 2A).

By blocking ERK, p38 MAPK, TGF-β/Smad and PI3K/AKT signalings via pre-treatment with their inhibitors PD98059, SB203580, SB431542, and LY294002, respectively for 1 h, we found that inhibition of p38 MAPK and TGF-β signalings rescued the down-regulation of VE-cadherin and increment of α-SMA when exposed to high concentration of Hcy, though the inhibition aggravated the expression level of PAI-1. Compared with Hcy-treated group, administration of LY294002 intervened in the up-regulation of α-SMA critically, as well as the expression of PAI-1 and vimentin. Abrogating the activation of ERK1/2 with PD98059 only showed a tendency for the recovery of the protein level of VE-cadherin, α-SMA and vimentin, indicating that ERK1/2 signaling might have a slight effect on the process of EndMT mediated by Hcy (Figure 2B). These results suggest that activation of TGF-β/Smad, PI3K/AKT and MAPK signalings participate in the process of Hcy-triggered EndMT.

### GT Relieved Hcy-Mediated Oxidative Stress

**FIGURE 3:**
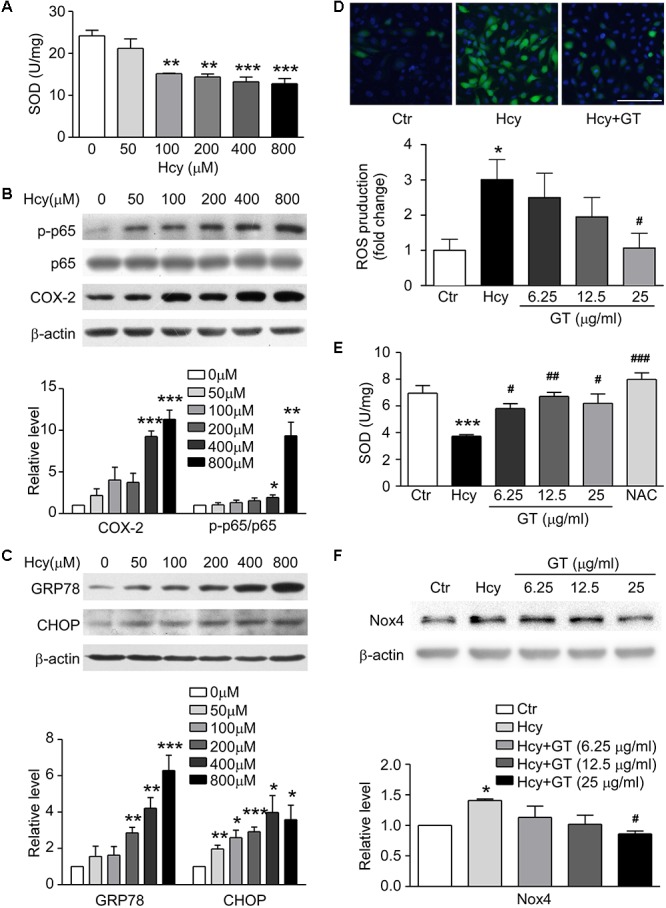
GT ameliorated Hcy-mediated oxidative stress. **(A)** SOD concentration in 0∼800 μM Hcy-treated groups. **(B)** Representative Western blotting (*up*) showing protein levels of p-NF-κB p65 and COX-2 in Hcy incubated groups. The quantification of protein levels is shown in lower panel. **(C)** Representative Western blotting (*up*) showing protein levels of GRP78 and CHOP in Hcy incubated groups. The quantification of protein levels is shown in lower panel. **(D)** Representative images showing the accumulation of ROS, the fluorescence intensity reflects the content of ROS (*top*). Bar represents 100 μm. The quantification of ROS production is shown in bottom panel. **(E)** The activity of SOD. **(F)** Representative Western blotting (*up*) showing the protein expression of Nox4 in the cells pre-incubated with GT. The quantification of protein levels is shown in lower panel. Data are mean ± SEM. *n* = 3; ^∗^*P* < 0.05; ^∗∗^*P* < 0.01; ^∗∗∗^*P* < 0.001 vs. control group. ^#^P < 0.05; ^##^P < 0.01; ^###^P < 0.001 vs. Hcy-treated group.

To ascertain the model of Hcy-induced endothelial dysfunction, we evaluated the level of oxidative stress, inflammation and ER stress. SOD was dose-dependently suppressed in Hcy-treated group (Figure 3A). The activation of NF-κB p65, subsequent up-regulation of COX-2 and substantiated ER stress activation were also found in Hcy infused BAECs (Figure 3B,C). These results confirm that oxidative stress works as an essential factor in the pathogenic mechanism of endothelial injury mediated by Hcy, which is partly involved in the pathogenic process of Hcy-induced inflammation and ER stress.

Following experimental results showed that Hcy-induced the accumulation of ROS could be eliminated by pretreatment of GT at 25 μg/ml (Figure 3D, top panel). The ROS production in Hcy-treated group was significantly increased, which was decreased with treatment of high-dosed GT (Figure 3D, bottom panel). NAC, a well-known anti-oxidant, almost restored the activity of SOD entirely. However, GT improved SOD activity at a lower dose (6.25 μg/ml) and reversed NADPH oxidase 4 (Nox4) expression at a higher dose (25 μg/ml). (Figure 3E,F). These data hint that GT attenuated Hcy-induced oxidative stress.

### GT Alleviated Hcy-Elicited EndMT via Canonical TGF-β/Smad and Non-smad Signalings

**FIGURE 4:**
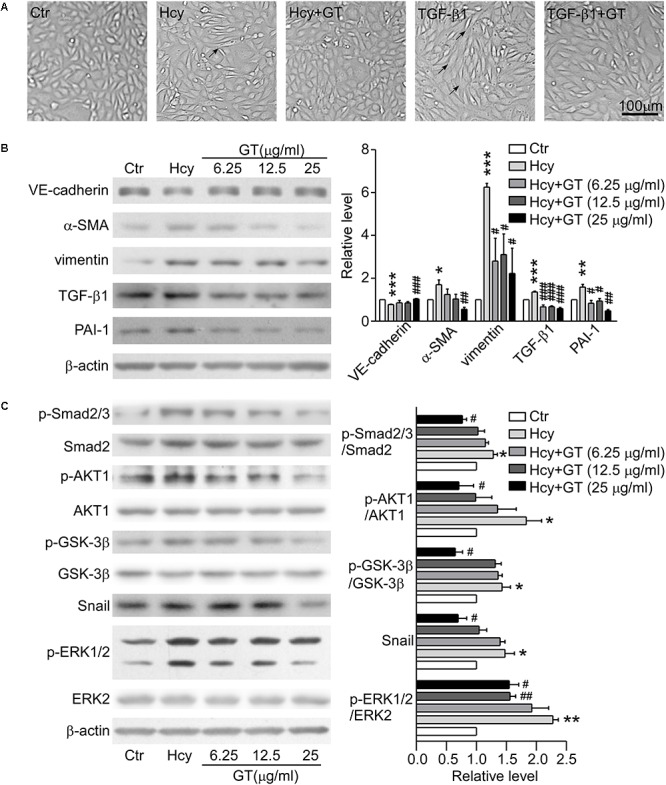
GT inhibited EndMT through regulating TGF-β/Smad, AKT/GSK-3β, and MAPK signalings. **(A)** Representative microscopy showing cell morphology of BAECs pre-incubated with or without 25 μg/ml GT for 1 h, and treated with Hcy or TGF-β1 or not for 48 h. The arrows indicate the typical cells. **(B)** Representative Western blotting (*left*) of the protein levels of VE-cadherin, α-SMA, vimentin, TGF-β1, and PAI-1 of cells after GT pre-incubation and Hcy treatment. The quantification of protein levels is shown in right panel. **(C)** Representative Western blotting (*left*) of the protein levels of p-Smad2/3, p-AKT, p-GSK-3β, Snail, p-ERK1/2 in cells pretreated with GT. The quantification of protein levels is shown in right panel. Data are mean ± SEM. *n* = 3; ^∗^*P* < 0.05; ^∗∗^*P* < 0.01; ^∗∗∗^*P* < 0.001 vs. control group. ^#^*P* < 0.05; ^##^*P* < 0.01; ^###^*P* < 0.001 vs. Hcy-treated group.

The morphologic images presented that the GT treatment rescued the abnormal morphologic changes of BAECs caused by Hcy and the formation of spindle-like shape induced by TGF-β1 (Figure 4A), suggesting that GT have an essential role in preventing EndMT. As Western blot results showed, pre-incubation with GT at 25 μg/ml prevented the drastic reduction of VE-cadherin and the augmentation of α-SMA in Hcy treated group, almost maintaining the same level with the control group. Meanwhile, the pre-treatment of GT at low dose (6.25 μg/ml) could lessen the expression of vimentin, PAI-1 and TGF-β1 without remarkable dose-dependent effect, compared to Hcy-treated group (Figure 4B). All of these implicated that GT repressed the process of Hcy-induced EndMT. In terms of cell migration assay results, we found that Hcy inhibited the migration of BAECs, but GT had minor effects on Hcy-impaired cellular motility (data not show). Thus, we postulated that GT might exhibit inhibitive effects on Hcy-mediated EndMT without improving its migratory ability.

Pre-incubation with 25 μg/ml GT, the protein levels of phosphorylated Smad2/3, ERK1/2, AKT, GSK-3β, and Snail in BAECs were dramatically decreased compared with Hcy group (Figure 4C). Taken together, our results suggest that canonical TGF-β/Smad and non-Smad dependent signalings are corporately involved in the protective effect of GT in EndMT.

## Discussion

**FIGURE 5:**
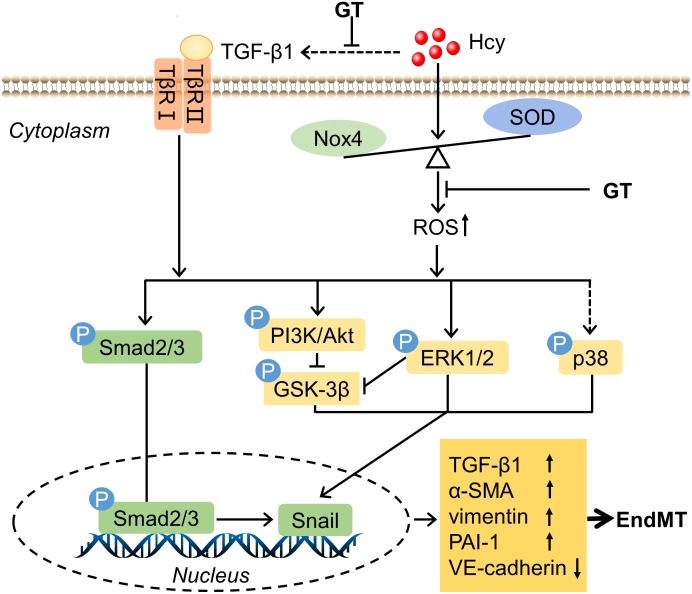
Schematic mechanism of endothelial cells dysfuntion exposure to HHcy. The detail see text. Arrows or lines represent the activation or inhibition relationship between two molecules. The dotted line represents that Hcy has indirect effect on TGF-β1 and little effect on the level of phosphorylation of p38.

The initial purpose of this study was to explore the mechanisms involved in HHcy mediated EC dysfunction comprehensively and to discover potential therapeutic drugs. Experimental data demonstrated that GT shed light on the treatment of HHcy-induced BAECs injury, which undergoes the process of EndMT and might be mediated by oxidative stress and TGF-β signaling pathway. The mechanisms we proposed in this study were illustrated in Figure 5.

Clinically, total concentration of serum Hcy above 15 μM is defined as HHcy, which has been classified to different levels corresponding to different clinical symptoms (Baloula et al., 2018). The concentration of Hcy we applied in this study correspond to the level of clinical intermediate (above 30 μM) and severe HHcy (above 100 μM), to which the experimental results are more applicable. In this study, we chose BAECs as the *in vitro* experimental model. Compared with other endothelial cell lines, BAECs exhibit stable phenotype and typical signaling pathways (Jin et al., 2012).

EndMT is a universal feature in organ development, regeneration and chronic fibrotic diseases. Similar with epithelial-mesenchymal transition (EMT) in its broadest sense, EndMT partially accounts for the fundamental mechanism of cardiovascular development and EC dysfunction (Evrard et al., 2016). In cardiovascular system, it has been reported that EndMT is involved in the progression of cardiac fibrosis in a mouse model of pressure overload and pulmonary hypertension (Zeisberg et al., 2007; Ranchoux et al., 2015). Recently, Evrard et al. firstly revealed that fibroblast-like cells driven by EndMT was a hallmarker in atherosclerotic lesions and exploited EndMT as a novel therapeutic target for atherosclerosis (Evrard et al., 2016).

Previous studies have documented that Hcy has profound biological effects on ECs both *in vitro* and *in vivo*. However, there is no research pointing out that EndMT could account for the pathological mechanism of HHcy-impaired ECs. Although Beard et al. reported that Hcy could regulate the phosphorylation of VE-cadherin and translocation of β-catenin to nucleus, their results showed that the expression of VE-cadherin had no evident alteration with 200 μM Hcy stimulation (Beard et al., 2012). In this study, we found significantly decreased level of VE-cadherin and increased α-SMA and vimentin levels after 400∼800 μM Hcy stimulation. Furthermore, only high doses of Hcy mediated the transition from endothelial cells to mesenchymal cells. The switch of cellular phenotype coincided with the transition of cell morphology from cobblestone to spindle. Strictly speaking, the cells occurring EndMT in the present study are transitioning cells that co-express EC and mesenchymal cell markers. Collectively, we speculate that EndMT might have a major role in HHcy-induced EC dysfunction and vascular remodeling.

Sporadic researches reported that HHcy induced EMT and ultimately gave rise to podocyte injury (Li et al., 2011; Zhang et al., 2011), but the underlying mechanism is still unknown. The regulation of EndMT related proteins is associated with the suppression and activation of pivotal transcriptional factors including Snail and its upstream intracellular signaling cascades including TGF-β/Smad, PI3K/AKT and MAPK (Xie et al., 2004; Gonzalez and Medici, 2014). TGF-β is the most well-known inducer participating in the initiation and progression of EndMT. Several evidences also strongly suggest that TGF-β/Smad signaling has a close relation with the expression level of PAI-1. Up-regulation of PAI-1 appears to be responsible for the generation of atherothrombosis, tissue fibrosis and aggressiveness of cancer (Wang et al., 2017). Although researchers have found that Hcy elevated the expression of TGF-β, the mechanisms below and the relation between HHcy and EndMT are still unclear. Making use of TGF-β RI kinase inhibitor VI SB431542, we found that the inhibition of TGF-β/Smad signaling could reverse the expression of VE-cadherin and α-SMA elicited by Hcy.

In addition, TGF-β is a potent activator of PI3K/AKT and MAPK signalings, which are independent of Smad signaling (Medici et al., 2011). Robust evidences indicate that activation of PI3K/AKT signaling is involved in the progress of fibrosis and its inhibition would exert anti-fibrotic effect (Zhao et al., 2015; Tsoyi et al., 2017). GSK-3β, which could be inactivated by the activation of PI3K/AKT signaling through phosphorylation modification, inhibits the stability and nuclear translocation of Snail. Snail is the zinc-finger binding transcription factor and could cooperate with other factors binding to promoter region of genes and regulates the activity of histone deacetylase to switch the layout of the cell-cell junction proteins, such as VE-cadherin (Oda et al., 1998). Zhang et al. (2016) has reported that arsenic trioxide induced cardiac fibrosis mediated by EndMT via activating AKT/GSK-3β/Snail pathway. The currently presented data showed that the activity of PI3K/AKT signaling was evoked within 1 h and GSK-3β was inhibited simultaneously leading to the translocation of Snail to nucleus. Pretreatment with PI3K inhibitor LY294002 partially abrogated the progression of EndMT, suggesting that PI3K/AKT is of great importance in the HHcy-induced EndMT.

Accumulating evidences have attested that MAPK also regulates the progression of EndMT through GSK-3β or transcription factors paralleled to our data (Li et al., 2017). The activation of ERK1/2 and p38 MAPK are important components in TGF-β mediated Smad-independent signaling. Inhibiting the activation of p38 rather than ERK1/2 MAPK by utilizing SB203580 in our study partly relieved the process of EndMT, despite the fact that Hcy did not affect the level of phosphorylated p38 obviously. The phenomenon suggests that activation of ERK1/2 is involved in Hcy-induced injury but plays a minor role in Hcy-mediated EndMT. Contrarily, the effect of SB203580 on HHcy-induced EndMT indicates p-p38 plays an important role in Hcy-induced EndMT, which might ascribe to p38/MAPK signaling dependent oxidative stress (Wang et al., 2012). Actually, the crosstalk among TGF-β/Smad, PI3K/AKT and ERK signalings is more intricate than we discussed here. Our data underscored that TGF-β/Smad, PI3K/AKT and MAPK signalings had canonical roles in the process of HHcy-induced EndMT.

It has been reported that the conversion of ECs into myofibrolasts through EndMT and the activation of TGF-β signaling could be driven by oxidative stress (Evrard et al., 2016). Increased ROS production and aberrant oxidase and anti-oxidase are main resources of oxidative stress in HHcy-induced EC injury. Among those, Nox4, an important producer of ROS in ECs, has been substantially reported upregulation with Hcy treatment (Liu et al., 2012). Although several researches showed that normal expression of Nox4 exerts protective effect on atherosclerosis, the increased level of Nox4 caused impaired ECs via inducing oxidative stress in HHcy (Liu et al., 2012; Kamat et al., 2015; Liao et al., 2018). Our results demonstrated that oxidative stress was triggered by regulating SOD2 and Nox4 activation and expression in HHcy condition, which might induce HHcy-mediated EndMT.

We identified that GT ameliorated oxidation state through recovering the antioxidant level and attenuated HHcy-induced cellular morphology change. Mechanistically, substantial ROS production from oxidative stress subsequently mediated the activation of Smad, AKT and MAPK signalings. Pretreatment of GT mitigated the abnormally altered expression of proteins and downstream signals related to EndMT. Altogether, GT showed potential to protect ECs from EndMT process via suppressing oxidative stress. Basing on experimental data, we postulate that GT exert potential cardiovascular-protective effect on HHcy-induced EndMT and endothelial injury.

Taken together, our study revealed the pathological mechanism of HHcy-induced endothelial dysfunction and that HHcy-induced EndMT via activating oxidative stress and TGF-β/Smad and non-Smad signalings. Additionally, our data support an insight that GT might have the therapeutic potential for HHcy-induced endothelial impairment via suppressing oxidative stress. Further researches are needed to reinforce these results by using animal models and explore the underlying mechanisms.

## Data Availability

The datasets generated for this study are available on request to the corresponding author.

## Author Contributions

HZ, BY, JH, and YS designed the experiments. JH, YS, YJ, and XG performed the research and analyzed the data. JH, YS, HZ, and BY interpreted the results. BY, HZ and JH prepared, drafted, and edited the manuscript. All authors commented and approved the final manuscript.

## Conflict of Interest Statement

The authors declare that the research was conducted in the absence of any commercial or financial relationships that could be construed as a potential conflict of interest.
